# The Contribution of Diseases to the Male-Female Disability-Survival Paradox in the Very Old: Results from the Newcastle 85+ Study

**DOI:** 10.1371/journal.pone.0088016

**Published:** 2014-02-07

**Authors:** Andrew Kingston, Karen Davies, Joanna Collerton, Louise Robinson, Rachel Duncan, John Bond, Thomas B. L. Kirkwood, Carol Jagger

**Affiliations:** 1 Institute for Ageing and Health, Newcastle University, Newcastle upon Tyne, United Kingdom; 2 Institute for Health and Society, Newcastle University, Newcastle upon Tyne, United Kingdom; Cardiff University, United Kingdom

## Abstract

**Background:**

Explanations for the male-female disability-survival paradox - that woman live longer than men but with more disability - include sex differences in diseases and their impact on disability and death. Less is known about the paradox in the very old. We examine sex differences in the presence and impact of disabling and fatal diseases accounting for the male-female disability-survival paradox in very late life.

**Methods:**

We use data from the Newcastle 85+ Study, a cohort of people born in 1921 and all recruited at age 85 in 2006. Participants underwent a health assessment (HA) at baseline, 18 months, 36 months, 60 months, and a review of their GP records (GPRR) at baseline and 36 months. We used multi-state modelling to assess the impact of specific diseases on disability and death. Disability (measured via ADLs/IADLs) was categorised as no disability (difficulty with 0 activities), or disabled (difficulty with one or more activities). Diseases were ascertained from review of general practice records and cognitive impairment which was defined as an sMMSE of 21 or less (from health assessment).

**Results:**

In participants who had complete HA and GPRR, women had more arthritis (RR = 1.2, 95% CI: 1.1–1.3) and hypertension (RR = 1.2, 95%CI 1.0–1.3), more disability, and were more likely disabled at all follow-ups. From multistate models, women with cerebrovascular disease (HR: 2.6, 95% CI: 2.1–3.4) or respiratory disease (HR: 2.0, 95% CI: 1.4–3.0) were more likely to become disabled than those without but this did not hold for men (sex difference p<0.01). Men were more likely to die from respiratory disease (HR: 2.2, 95% CI: 1.8–2.8) but this did not hold for women (p = 0.002).

**Conclusion:**

The disability-survival paradox was still evident at age 85 and appears due to sex differences in the types of diseases and their impact on the disability pathway.

## Introduction

Women live longer than men on average, but their longer life expectancy is accompanied by more years with disability, both in absolute terms and as a proportion of remaining life [1].

Understanding the basis of this “disability-survival paradox” [2], [3] is important for addressing the different health challenges faced by very old men and women, the fastest growing age group in many countries [4], and could inform more effective clinical practice. The paradox may derive from intrinsic differences (biological, social or behavioural) between men and women [3], [5], [6]. Women are reported to have a greater number of acute and non-fatal chronic diseases, whereas men have fewer diseases in total but more of these are life-threatening [5], [7], [8]. A potential basis for a biological difference between men and women is the actions of sex hormones. Female sex hormones bring benefits for women by modulating lipid levels, and hence cardiovascular risk, and by affecting the immune response [5]. A recent report describes longer lifespans for Korean eunuchs than intact men, which is consistent with the idea that male sex hormones, notably testosterone, may be a risk factor for earlier mortality [9], notwithstanding the limitations of such historical studies. Behavioural differences between women and men include their perception of symptoms and readiness to consult with healthcare professionals. Sex differences in physician diagnostic patterns and self-reporting of disease may also contribute [10]. It is also possible that the progression of disease to disability may be more marked for women than men, especially if women are under-treated for some conditions [11]. Men’s higher mortality may also result from a greater severity of disease, which is inadequately captured in analyses based on the simple presence/absence of a condition. The contribution of such distortion has previously been reported to be small but it cannot altogether be discounted [2].

The Newcastle 85+ Study is a population-based longitudinal study of health and ageing in the very old. The comprehensive, multidimensional health assessment performed in this study, combined with the high level of success in recruiting from this age group [12], has provided a rich resource from which we can determine whether there are sex differences in the impact of specific diseases on disability and survival beyond age 85. More specifically we have examined the disability-survival paradox with a single hypothesis in mind: that the gender disparity in mortality and disability is driven by sex differences in the type of disease and their impact on disability.

## Methods

### Recruitment and Study Protocol

The sampling frame for the Newcastle 85+ Study comprised all surviving adults born in 1921, who turned 85 in 2006 when the study commenced, and who were permanently registered with a participating general practice in Newcastle or North Tyneside NHS Primary Care Trusts in North-East England. Full details of study design and participant recruitment have been reported [12]–[14]. At baseline, participants underwent a detailed multidimensional health assessment conducted by a trained research nurse in their usual place of residence (own home or institution). Data on diagnosed diseases (with date of first diagnosis) and prescribed medication were obtained from participants’ general practice (GP) medical records. Following baseline assessment, participants were re-assessed at 18, 36 and 60 months.

### Disability Measures

**Figure 1:**
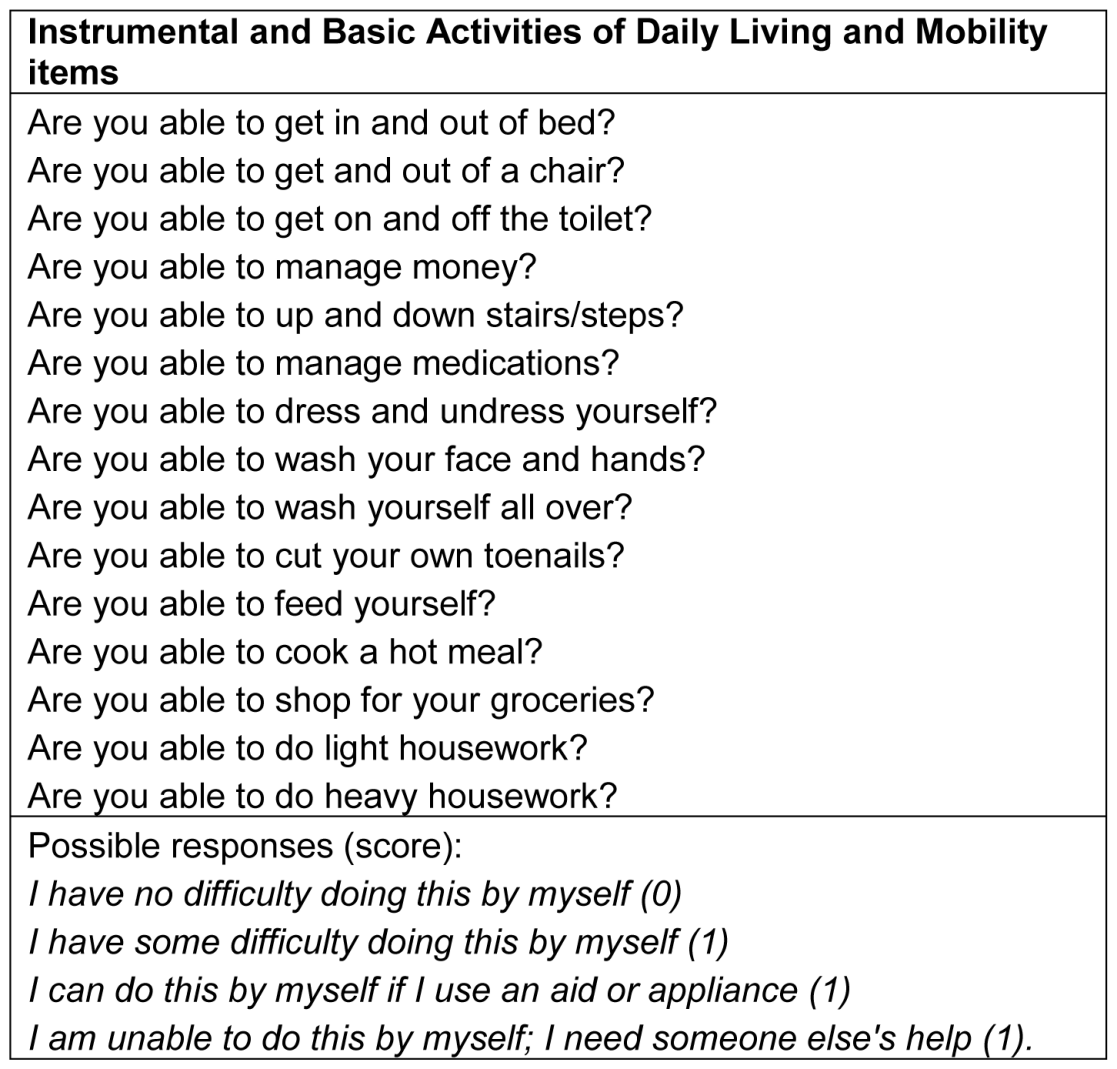
Intrumental and Basic Activites of Daily Living (IADLs, BADLs) and mobility items included in the disability score with possible repsonses.

At baseline and follow-up assessments, participants were asked about their ability to perform 15 activities comprising Instrumental and Basic Activities of Daily Living (IADLs, BADLs) and mobility items (Figure 1) [15]; these were taken predominantly from the Groningen Activity Restriction Scale [16]. As loss of ability for individual items formed a single hierarchy, similar for men and women [17], we calculated a disability score scoring 0 for each item reported to be performed without difficulty and 1 for each item performed with difficulty (maximum score 15). Participants were classified as having disability (difficulty with one or more items) or no disability (difficulty with no items). The association between self-reported performance in mobility items and an objectively measured timed-up-and-go (TUG) [18] test was high, and similar in both men and women [15].

### Disease Status

Disease status at baseline was ascertained predominantly from GP medical records; data extraction was conducted by trained research nurses following a standard protocol. Inter-rater reliability assessment demonstrated at least moderate agreement between the nurses for all diseases [13]. In the UK, patients are registered with a single general practice which acts as a gatekeeper to secondary care and receives details of all hospital admissions and outpatient attendances. The review of general practice records included hospital correspondence to ensure that all pre-existing diagnoses were extracted irrespective of where the diagnosis was made (from both paper and electronic formats). The only exception to ascertainment from GP records was for cognitive impairment, which we defined by a Standardised Mini-Mental State Examination (SMMSE) score of 21 or below [19]; SMMSE was conducted as part of the participant health assessment.

**Figure 2:**
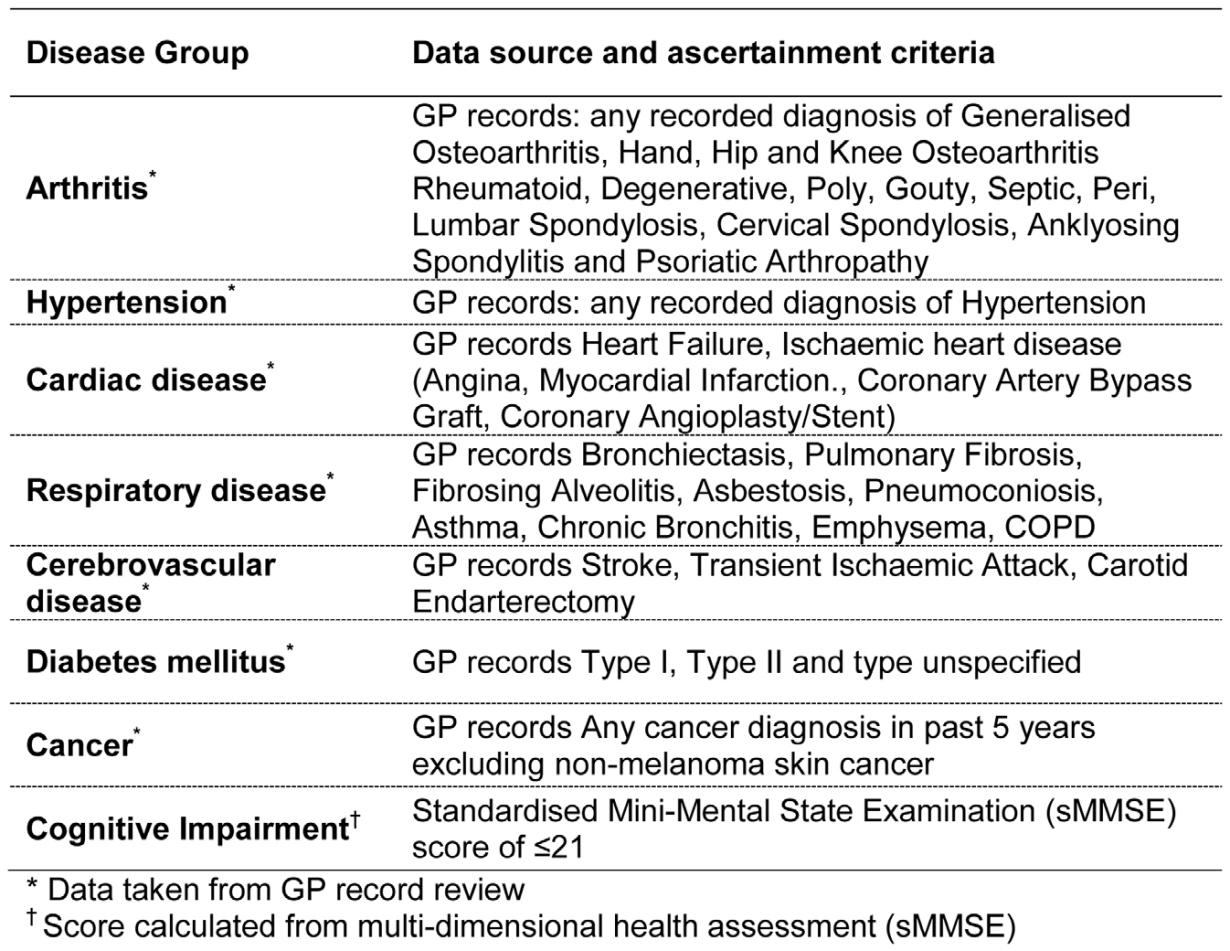
Conditions examined with data sources and ascertainement criteria.

For the purpose of this analysis, we focused on the eight most prevalent diseases in our cohort; in some cases we grouped multiple conditions into a category (e.g. all arthritic diseases) whilst other diseases were retained as single entities (e.g. hypertension) (Figure 2). For each participant we calculated a disease count (maximum score 8). A further review of GP records was conducted at 36 months and the SMMSE was re-administered at wave three and four. Individual diseases and conditions and the disease count were therefore updated and included in the models as time-varying covariates.

### Mortality

Participants’ medical records were flagged with the National and Social Care Information Service to provide date and cause of death. Survival time was calculated from date of baseline health assessment to date of death or censored at 1^st^ September 2012.

### Statistical Methods

Sex differences in the prevalence of each disease were analysed by Generalised Linear Models and presented as relative risk (RR) with 95% confidence intervals. We assessed gender differences in IMD and education at baseline by ordinal logistic regression and presented them as odds ratios (OR) with 95% confidence intervals. Sex differences in the level of disability at baseline were analysed by Tobit regression [20] to account for the ‘floor effects’ in the disability score and adjusted for: years of full-time education (0–9 years/10–11 years/12+ years); Index of Multiple Deprivation (IMD), an area level measure of socio-economic disadvantage (categorised as <20^th^ centile, 20–80^th^ centile, >80^th^ centile) [21]; and disease count. A similar approach was used to compare sex differences in baseline disability associated with each specific disease with adjustment for residual disease count (disease count excluding the disease of interest), IMD and education. We present the difference in disability scores with 95% confidence interval.

To assess the contribution of specific diseases to transitions to and from disability and to death over five years, we fitted a multistate model in continuous time with three states: no disability, disability, and death (absorbing state). We used this model to estimate the instantaneous rate of transition between the states (presented as hazard rates with 95% confidence intervals and mean time in the state) making the assumption that transition from no disability to death was via disability. Models were fitted with each disease individually and then adjusted for the residual disease count. Models were further adjusted for IMD and education. Since participants were all born in 1921 (and all aged 85 at baseline) we did not adjust for age in any models. Analyses were carried out in R version 2.9.1 using the msm package [22].

### Ethical Issues

Ethical approval was obtained from Newcastle & North Tyneside Local Research Ethics Committee One. Written informed consent was obtained from participants; where people lacked capacity to consent, for example because of cognitive impairment, a formal written opinion was sought from a consultee, usually a relative or carer.

## Results

### Selection and Key Characteristics of Study Population

At baseline, data from both participant assessment and GP records was available for 854 participants; 2 people subsequently withdrew and requested all data destroyed; 2 did not have complete data on disability and 7 did not have complete GP records. The remaining 843 had complete data for disability and disease status and formed the sample for analysis. The majority (61.9%, n = 522) were female. Of the key characteristics at baseline, only level of deprivation showed a significant sex difference with women having higher levels of disadvantage than men (OR F:M = 1.3, 95% CI: 1.0–1.7) (Table 1).

**Table 1:** Study population key characteristics.

	Male (n = 321)	Female (n = 522)	All (n = 843)	P-Value§	Sex Difference (95% CI)
**Education (years)**					
0–9	61.99 (199)	65.71 (343)	64.29 (542)	0.288	0.86 (0.65–1.14)*
10–11	24.92 (80)	21.65 (113)	22.89 (193)		
12+	13.08 (42)	12.64 (66)	12.81 (108)		
**Area deprivation (measured by IMD)**					
Low (<25 centile)	29.91 (96)	21.65 (113)	24.79 (209)	0.031	1.33 (1.02–1.74)*
Middle (25–75 centile)	47.04 (151)	52.87 (276)	50.65 (427)		
High (>75 centile)	23.05 (74)	25.48 (133)	24.56 (207)		
**Disease at baseline**					
Arthritis	60.44 (194)	71.84 (375)	67.50 (569)	0.001	1.19 (1.07–1.32)†
Hypertension	52.34 (168)	59.96 (313)	57.06 (481)	0.034	1.15 (1.01–1.30)†
Cardiac disease	42.06 (135)	35.63 (186)	38.08 (321)	0.060	0.85 (0.71–1.01)†
Cerebrovascular disease	24.61 (79)	18.97 (99)	21.12 (178)	0.050	0.77 (0.59–1.00)†
Respiratory disease	22.43 (72)	22.61 (118)	22.54 (190)	0.953	1.01 (0.78–1.30)†
Diabetes mellitus	14.33 (46)	12.64 (66)	13.29 (112)	0.483	0.88 (0.62–1.25)†
Cognitive impairment	10.28 (33)	14.56 (76)	12.93 (109)	0.076	1.42 (0.96–2.08)†
Cancer	8.10 (26)	5.36 (28)	6.41 (54)	0.104	0.65 (0.39–1.09)†
**Disease count median (mean(sd))**	2.4 (1.3)	2.4 (1.3)	2.4 (1.3)	0.680	0.06 (−0.11–0.24)‡

*Ordinal logistic regression – Odds ratio - men: women.

† Generalised linear model - Relative Risk - men: women.

‡ T-test – difference in disease count - men: women.

§ P-value for gender difference.

### Disease and Disability Prevalence at Baseline

At baseline, women were more likely to have a diagnosis of arthritis (RR: 1.2, 95% CI: 1.1–1.3) or hypertension (RR: 1.2, 95% CI: 1.0–1.3) and less likely to have a diagnosis of cerebrovascular disease (CVD) (RR: 0.8, 95% CI: 0.6–1.0) (Table 1). There was no evidence of any sex difference in the total number of diseases (p = 0.68) or in disease duration (time since first diagnosis) for any disease. Table S1 details the disease duration by gender.

Sex differences in the baseline disability score, between participants with and without specific diseases at baseline, demonstrated a broadly similar disabling impact for men and women (Table 2). For both sexes, cognitive impairment (SMMSE≤21) conferred the greatest disability, by approximately 7 points compared to those cognitively intact, then CVD with a difference in disability score of 3 points in women and 2 in men. Compared to men, women had a significantly greater disability score at baseline for all diseases except cognitive impairment and cancer where no difference was evident (Table 2). Where disease was not present levels of disability remained higher for women than men across all disease groups even after adjustment for potential confounders (deprivation, education and residual disease count).

**Table 2:** Disability by disease status at baseline.

	Disability Score - Median (IQR)							
	Men			Women			Sex difference in disability score†	
	With disease	Without disease	Disability score difference (95% CI)*	With disease	Without disease	Disability score difference (95% CI)*	With disease	Without disease
Arthritis	2 (1–5)	1 (0–4)	1.55 (0.27,2.83)	3(1–7)	2(0–5)	1.68 (0.68,2.67)	1.82 (0.94,2.70)	1.78 (0.32,3.23)
Hypertension	1 (0–4)	2 (0–6)	−0.92 (−2.16,0.32)	3(1–7)	3(1–7)	0.01 (−0.90,0.92)	2.37 (1.39,3.35)	1.51 (0.34,2.69)
Cardiac Disease	2 (0–5)	1 (0–5)	−0.17 (−1.43,1.09)	4(2–8)	3(1–6)	1.26 (0.34,2.18)	2.80 (1.69,3.90)	1.46 (0.45,2.48)
CVD	2 (1–6)	1 (0–4)	2.15 (0.75,3.56)	6(3–11)	3(1–6)	3.10 (2.01,4.20)	2.99 (1.28,4.69)	1.86 (1.05,2.67)
Respiratory disease	2 (0–5)	1 (0–4)	0.69 (−0.79,2.17)	4(2–8)	3(1–6)	0.94 (−0.11,2.00)	2.12 (0.68,3.56)	1.92 (1.05,2.80)
Diabetes	2.5 (0–6)	1 (0–4)	0.64 (−1.12,2.41)	4(3–7)	3(1–7)	1.18 (−0.14,2.51)	2.37 (0.53,4.21)	1.91 (1.09,2.73)
Cognitive Impairment	11 (6–13)	1 (0–3)	7.85 (6.14,9.56)	9(5.5–13.5)	3(1–5)	6.28 (5.15,7.40)	0.37 (−2.00,2.74)	1.79 (1.11,2.46)
Cancer	2 (1–3)	1 (0–5)	0.72 (−1.46,2.90)	2.5(1–7)	3(1–7)	−0.23 (−2.22,1.76)	1.19 (−1.39,3.77)	2.12 (1.33,2.90)

*Tobit regression: difference in disability score for those with and without disease.

† Tobit regression: sex difference in disability score for those with and without disease: women compared to men.

Overall, women reported difficulty with almost two more activities on average than men (difference in mean disability score: 2.0, 95% CI: 1.2–2.7), even after adjusting for education, deprivation and disease count (Table 3).

**Table 3:** Disability by gender and participation status at baseline and follow-up waves.

	Disability Score at interview			Disability Score at previous interview		
	Median (IQR)			Median (IQR)		
	Men	Women	Sex difference*	Men	Women	Sex difference*
**Baseline** **(n = 843)**	0 (1–5)	3 (1–7)	1.97 (1.21,2.72)	–	–	–
**18 months (Wave 2)**						
Participant (n = 626)	2 (1–6)	4.5 (2–9)	1.90 (1.11–2.70)	1 (0–4)	3 (1–5.5)	1.79 (1.01–2.58)
Died before W2 (n = 66)	–	–	–	6 (1–12)	9 (4–14)	3.53 (0.29–7.35)
Withdrawn before W2 (n = 151)	–	–	–	2 (1–6)	5 (2–9)	2.54 (0.85–4.24)
**36 months (Wave 3)**						
Participant (n = 482)	4 (1–8)	5 (3–9)	1.33 (0.41–2.26)	2 (1–6)	4 (2–7)	1.76 (0.91–2.61)
Died before W3 (n = 52)	–	–	–	5 (2–8)	11 (3–14)	4.23 (1.06–7.40)
Withdrawn before W3 (n = 92)	–	–	–	3 (1–8)	7 (4–12)	2.29 (0.20–4.39)
**60 months (Wave 4)**						
Participant (n = 342)	4 (1–7)	5 (3–9)	1.74 (0.60–2.87)	3 (1–7)	4 (3–7)	1.04 (0.55–2.02)
Died before W4 (n = 81)	–	–	–	5 (1–10)	11 (5–13)	2.98 (0.51–5.44)
Withdrawn before (n = 59)	–	–	–	5 (1.5–10.5)	7 (3–11)	2.20 (−0.95–5.35)

*Tobit regression sex difference compared women to men.

### Impact of Disease on Transitions to Disability and Death over 5 years

At each of the 18, 36 and 60 month follow-ups, over 70% of participants remained in the study (18 month: n = 626; 36 months: n = 482; 60 months: n = 342), 7–12% withdrew (18 month: n = 151; 36 months: n = 51; 60 months: n = 59) whilst around 15% died (18 month: n = 66; 36 months: n = 92; 60 months: n = 81) (Table 3).

Higher levels of disability found in women at baseline were also manifest at subsequent follow-up waves (Table 3). This pattern was unlikely to result from men being less likely to report difficulty in performance than women, as the relationship between reported performance on mobility items and the objectively measured TUG test were similar in men and women, at baseline and subsequent waves. Compared to men, women had higher levels of disability in the interview prior to dropout, whether dropout was due to death or withdrawal (Table 3).

We used multistate models to explore sex differences in the progression to disability and death for each disease after adjustment for residual disease count, education and deprivation (Table 4). Diabetes conferred the highest risk of incident disability in men (HR: 3.0, 95% CI: 2.4–3.8) and women (HR: 1.7, 95% CI: 1.3–2.2) (Table 4). Despite the prevalence of arthritis being highest in women, its impact on incident disability was greater for men (HR: 1.7, 95% CI: 1.2–2.5) than women (HR: 1.2, 95% CI: 1.0–1.5) but arthritis conferred a significantly increased risk of becoming disabled in both sexes. Both men (HR: 1.6, 95% CI: 1.3–1.9) and women (HR: 2.4, 95% CI: 1.9–3.0) with cardiac disease had significantly greater risk of incident disability but the risk was higher for women (p = 0.003). A greater risk of incident disability was also evident for cognitive impairment (men HR: 1.3, 95% CI: 1.1–1.6; women HR: 1.7, 95% CI: 1.0–2.9). On the other hand CVD (HR: 2.6, 95% CI: 2.1–3.4) and respiratory disease (HR: 2.0, 95% CI: 1.4–3.0) increased the risk of incident disability for women only.

**Table 4:** Hazard rates (HR) and 95% confidence intervals (95% CI) for transitions between disability states and death adjusted for comorbidity, deprivation, and education.

	Men	Women	Sex Difference *p*-value
**Incident Disability**	Referent*	1.26 (1.12–1.41)	0.041
Arthritis	1.72 (1.19–2.48)	1.23(1.02–1.49)	0.942
Hypertension	0.87 (0.48–1.58)	1.09(0.56–2.12)	0.315
Cardiac Disease	1.60 (1.32–1.93)	2.39(1.92–2.97)	0.003
Cerebrovascular Disease	1.11 (0.76–1.63)	2.63(2.06–3.35)	0.000
Respiratory disease	0.98 (0.74–1.29)	2.02(1.35–3.01)	0.002
Diabetes	3.03 (2.43–3.79)	1.67(1.26–2.22)	0.001
Cognitive Impairment	1.31 (1.06–1.62)	1.71 (1.02–2.86)	0.174
Cancer	0.84 (0.29–2.42)	1.85(0.71–4.80)	0.139
**Death from no disability**	Referent*	0.89(0.73–1.08)	0.216
Arthritis	0.70 (0.33–1.50)	0.99(0.47–2.13)	0.260
Hypertension	0.62 (0.28–1.34)	1.01(0.44–2.31)	0.200
Cardiac Disease	1.42 (1.17–1.73)	1.48 (0.97–2.26)	0.569
Cerebrovascular Disease	1.31 (1.09–1.57)	1.00(0.59–1.70)	0.173
Respiratory disease	2.16 (1.67–2.79)	1.00(0.49–2.04)	0.046
Diabetes	1.20 (0.88–1.62)	1.01(0.24–4.17)	0.592
Cognitive Impairment	1.68 (1.41–2.01)	1.38(1.01–1.89)	0.857
Cancer	4.10 (2.35–7.13)	1.10(0.65–1.86)	0.001
**Disability recovery**	Referent*	0.96 (0.80–1.15)	0.328
Arthritis	0.73 (0.42–1.28)	0.96(0.72–1.27)	0.196
Hypertension	1.63 (0.94–2.81)	0.79(0.30–2.08)	0.899
Cardiac Disease	0.94 (0.57–1.56)	0.96(0.49–1.88)	0.481
Cerebrovascular Disease	1.63 (0.50–5.38)	0.41(0.03–5.03)	0.837
Respiratory disease	1.07 (0.58–1.97)	0.82(0.26–2.66)	0.651
Diabetes	0.85 (0.28–2.57)	0.89(0.54–1.47)	0.470
Cognitive Impairment	0.17 (0.09–0.31)	0.17(0.03–0.88)	0.500
Cancer	0.94 (0.67–1.33)	0.90(0.53–1.54)	0.547
**Death from disabled**	Referent*	0.84 (0.72–0.98)	0.042
Arthritis	0.84 (0.61–1.17)	0.92(0.51–1.66)	0.403
Hypertension	0.81 (0.59–1.10)	1.08(0.76–1.52)	0.111
Cardiac Disease	1.46 (1.21–1.77)	1.40(1.11–1.78)	0.601
Cerebrovascular Disease	1.09 (0.62–1.92)	1.36(1.03–1.80)	0.244
Respiratory disease	1.39 (0.82–2.35)	1.42(1.05–1.92)	0.474
Diabetes	1.27 (0.87–1.87)	1.11(0.76–1.63)	0.693
Cognitive Impairment	2.49 (1.76–3.54)	2.62(1.81–3.78)	0.428
Cancer	1.43 (0.92–2.22)	1.51(1.10–2.08)	0.416

*Referent category for assessing gender difference adjusted for full disease count.

Significant sex differences in the risk of death for those without disability were observed only for cancer (men: HR: 4.1, 95% CI 2.4–7.1; women: HR: 1.1, 95% CI: 0.7–1.9) and respiratory disease (men: HR: 2.2, 95% CI 1.7–2.8; women: HR: 1.0, 95% CI: 0.5–2.0) with male participants being at increased risk compared to their female counterparts (Table 4). Men with cardiac disease (HR: 1.4, 95% CI: 1.2–1.8) or CVD (HR: 1.3, 95% CI: 1.1–1.6) were at increased risk of death from a non-disabled state but this did not differ significantly from their female equivalents. The risk of death from a non-disabled state was significantly increased for both men and women with cognitive impairment (men: HR: 1.7, 95% CI: 1.4–2.0; women HR: 1.4, 95% CI: 1.0–1.9).

Recovery from disability was rare and lowest for participants with cognitive impairment though similarly for men (HR: 0.2, 95% CI: 0.1–0.3) and women (HR: 0.2, 95% CI: 0.03–0.9) and in both cases significantly less likely compared to participants without cognitive impairment (Table 4).

Hazard ratios for the risk of death once disabled were of similar magnitude for those with cognitive impairment (men HR: 2.5, 95% CI: 1.8–3.5; women HR 2.6, 95% CI: 1.8–3.8) and cardiac disease (men HR: 1.5, 95% CI: 1.2–1.8; women HR: 1.4, 95% CI: 1.1–1.8). CVD increased the risk of death once disabled for women only (HR: 1.4, 95% CI: 1.0–1.8) as did respiratory disease (HR: 1.4, 95% CI: 1.1–1.9) and cancer (HR: 1.5, 95% CI: 1.1–2.1).

**Figure 3:**
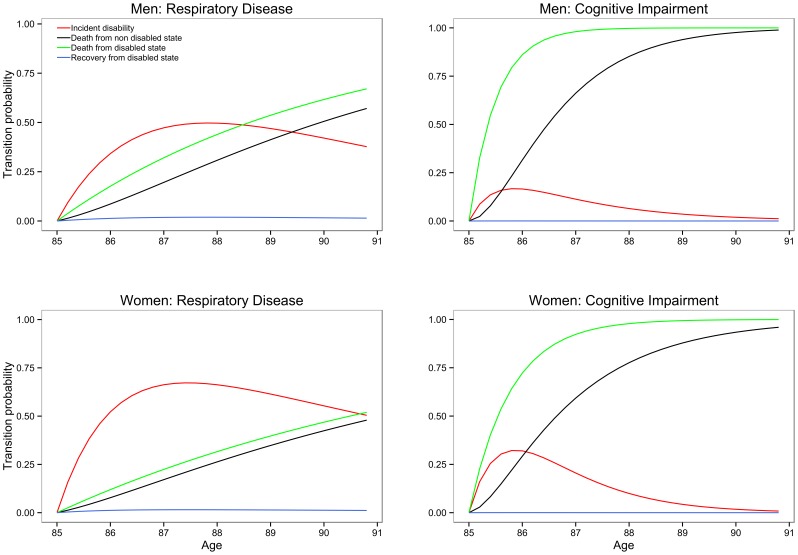
Transition probabilties for two diseases by gender.

The varied way in which different diseases impact on transitions to and from disability and to death for men and women is illustrated in Figure 3 for two diseases: cognitive impairment and respiratory disease. Cognitive impairment confers a very high risk of disability with little chance of recovery from disability and a high risk of death, but little difference exists between men and women. Respiratory disease on the other hand is significantly disabling only in women and has a higher risk of death for men initially disability free and women initially disabled.

### Mean Time with and without Disability

Overall, and regardless of disease status, more years are spent after age 85 with disability than without for both men and women, with women spending 2.2 years more on average with disability and 0.5 years less without disability than men (Table 5).

**Table 5:** Mean sojourn times in state (years) by disease group.

	Without disability		With Disability	
	Without disease	With disease	Without disease	With disease
	**Women**			
	1.31		6.44	
Arthritis	1.47	1.20	6.12	6.51
Hypertension	1.37	1.26	5.97	6.70
Cardiac Disease	1.53	0.64	6.95	5.29
CVD	1.38	0.53	6.61	5.67
Respiratory disease	1.46	0.72	6.86	5.26
Diabetes	3.46	2.07	4.36	5.20
Cognitive Impairment	3.58	2.23	4.64	3.04
Cancer	1.36	0.73	6.58	4.67
	**Men**			
	1.82		4.20	
Arthritis	2.52	1.60	4.09	5.11
Hypertension	1.87	2.22	4.34	5.00
Cardiac Disease	2.46	1.57	5.44	3.88
CVD	2.12	1.92	4.85	4.25
Respiratory disease	2.15	1.56	4.74	3.89
Diabetes	2.28	0.81	4.86	3.97
Cognitive Impairment	2.04	3.36	5.21	2.41
Cancer	2.04	1.81	4.79	3.43

For hypertension and arthritis, men and women spent longer with disability for both sexes than those without the disease, reflecting the low fatality and disabling effects of these conditions. This was additionally true for women with diabetes. For any of the diseases examined, women with the disease spent fewer years without disability compared to women without the disease. This was also broadly similar for men although men with hypertension spent more years (0.35 years) disability-free than men without hypertension and men with cognitive impairment spent 1.32 years longer disability-free than men without cognitive impairment.

## Discussion

The goal of this paper was to determine potential reasons for the male-female disability survival paradox in the very old, in particular the role of specific diseases on disability and mortality. We approached the investigation with a single question in mind: was the mortality and disability difference between men and women primarily driven by sex variation in the type and impact of diseases [23].

We found that at age 85 women already had a higher prevalence of disability than men and were more likely to have arthritis and hypertension. Despite women having marginally less CVD and cardiac disease than men at age 85, these conditions resulted in higher disability scores in women at baseline and prospectively were more likely to be disabling in women. Respiratory disease was also significantly more disabling in women than men although prevalence at age 85 was similar for men and women. Thus from age 85 women spent longer with disability than men overall (on average 2.2 years) and by disease. No sex differences were found in disease-specific recovery from disability and only for cancer was there a sex difference in disease-specific risk of death. These findings were not a consequence of differences in education, current socio-economic status (as measured by IMD) or the presence of comorbidity. Neither were they due to men with more disability dying or withdrawing between assessments as comparison of disability scores in the interview prior to death or withdrawal again demonstrated excess disability in women over men. Thus we suggest that the disability-survival paradox in the very old is at least partly due to sex differences in the type and disabling impacts of diseases.

Very old men have a marginally greater prevalence of diseases which are more likely to kill (cardiac disease, CVD and cancer), and women a higher prevalence of the chronic diseases (arthritis and hypertension). Nevertheless, this difference did not always translate to increased disability incidence or death. Arthritis was more disabling for men and cardiac disease for women, whilst cancer and cognitive impairment were the most fatal diseases for women (after becoming disabled). Earlier studies, mainly in younger age groups, have found that women are significantly more likely than men of the same age to have disabling rather than fatal diseases [3], [5], [6]. We can confirm that very old women (85+) do have a significantly greater prevalence of chronic diseases (arthritis, hypertension). Whilst men of the same age had greater prevalence of fatal diseases (cardiac disease, CVD and cancer) than women the differences were not statistically significantly different. Furthermore, the presence of certain fatal diseases increased the likelihood of incident disability more for women than men and increased the likelihood of death from a non-disabled state more for men than women.

Longitudinally, the presence of arthritis, cardiac disease, diabetes and cognitive impairment was significantly disabling for both sexes and CVD and respiratory disease for women only. This is generally consistent with previous findings [24] although in our study CVD and respiratory disease were significantly disabling in women only, perhaps caused by slight differences in criteria considered for a CVD diagnosis that may favour women compared to other studies. Furthermore we found cardiac disease, CVD, and respiratory disease to be more disabling for women compared with men whilst cognitive impairment was similarly disabling in both sexes. Diabetes was also disabling for both sexes, however the impact was noticeably worse for men (HR: 3.0, 95% CI: 2.4–3.8) compared to women (HR: 1.7, 95% CI: 1.3–2.2) (p = 0.001).

### Implications for Clinical Practice

Few recoveries from disability were observed regardless of the presence or absence of specific diseases, and were particularly low in the presence of cognitive impairment. Indeed cognitive impairment was detrimental for disability incidence, recovery and mortality for both sexes as previously found [24]–[26]. Gill *et al* analysed trajectories of disability on a monthly basis [27] and showed that among people in advanced stages of dementia, 67.9% had persistent severe disability. Our results similarly showed that those who became cognitively impaired moved swiftly into disability and then death (figure 3). This adds to the already strong argument for better preventative care in those at higher risk of developing cognitive impairment, especially as projection modelling from large cohort studies has predicted the link between ageing populations, dementia and disability. Despite concerns about screening for mild cognitive impairment and dementia [28] the very old should perhaps be considered a ‘high risk’ population worthy of targeted case finding, in view of our findings and the fast progression from cognitive impairment to disability [29]. Furthermore, cognitive impairment was shown to be the most disabling disease, confirming its importance as a primary determinant of disability [30]. Using the same measure of cognitive impairment, a UK study reported that its elimination would save around 3.5 total life years and 4.3 years free of disability at age 65, and therefore with a greater impact on disability. In terms of slowing the deterioration in global functioning of people with dementia, anti-cholinesterase drugs have been shown to be cost-effective in both the early and moderate stages of Alzheimer’s disease [31], with recent evidence showing benefit also in advanced stages [32]. Evidence is growing around the effectiveness of some non-drug interventions, such as cognitive stimulation in routine dementia care, although there remains uncertainty about the most cost-effective way of delivering such interventions in practice. There is also an increasingly strong argument for better preventative care in those at higher risk of developing dementia. Based on our findings this may be particularly the case for women. More timely diagnoses would lead to earlier intervention which may delay the onset of significant disability from the moderate and advanced stages of cognitive impairment.

Greater fatality in men with respiratory disease (with no disability) may explain the greater disabling impact observed in women, by means of accelerated transit through the disablement process to death for men, a process for which we found no evidence in women. However the greater disabling impact of cardiac disease and CVD in women cannot be explained this way. Global estimates of the prevalence of angina have been shown to be significantly greater for women but men diagnosed with the same disease have an excess MI [33]. Whilst little is known about the etiological causes it could go some way to explain our results since, if men diagnosed with angina are at greater risk of MI [34] compared with their female counterparts, they may be more likely to die before we could detect disability. Our results indicate that cardiac disease is disabling for both men and women but such men have increased mortality whilst this is not true for women. However, once disabled, men and women with cardiac disease are more likely to die than their counterparts without the disease. This suggests that care packages for those with cardiac disease should be tailored towards reducing mortality in men and reducing disability in both sexes.

### Strengths and Limitations

Our study has strengths and limitations mainly in regard to measurement of disease and disability. That sex differences in self-reported disease were avoided in our study, as disease was ascertained from general practice records, can be viewed as both a strength and a limitation. Whilst in general women are more likely to consult health professionals than men, general practitioner consultation rates among the very old are high overall, and in our study did not differ between men and women. However we had previously found that women had lower rates of outpatient attendance than men [13]. Our diagnosis of disease was an ‘ever’ diagnosis (with the exception of cancer which was within the previous five years only) and we did not have information on disease severity, though analysis of disease duration showed no significant sex difference. Given disease was ascertained from general practice records, there may have been sex differences in undiagnosed disease. Through further measurements in the health assessment we have explored rates of undiagnosed disease for diabetes and hypertension and found rates of undiagnosed diabetes were low with no sex difference and, though the prevalence of undiagnosed hypertension was high (based on a single-occasion blood pressure measurement) again no sex difference was detected [13]. A further strength is that we investigated two levels of disability, milder and more severe, but this did not alter the conclusions. Finally, self-report of mobility items included in the disability score were highly correlated with objective measures (timed up-and-go) similarly in men and women.

### Conclusion

Once health deteriorates, mortality rates increase more for men than women [35] and this is revealed in our results where the impacts of certain diseases are disabling for women but detrimental to survival for men. Men who encounter diseases which increase mortality could be accelerated through the disability pathway [36] (and ultimately death). However the time intervals of our study are too wide to capture this potential, accelerated transit. Nevertheless, if, as posited, men traverse the disablement process faster than women once they encounter disease, it would further suggest that they do not just ‘age faster’ biologically than women [37]. We suggest that our results point to two different biological mechanisms driving the male-female disability-survival paradox: the sex difference is driven by a female heath disadvantage as well as being accompanied by a female mortality advantage, consistent with other findings [38]–[40]. Our results suggest that the potential acceleration through the disablement pathway for men may be caused by the gender-specific effect of disease (and severity) and/or its potential subsequent sequelae. Alternatively, it could be that men and women follow different routes through the disability pathway and thus women will, intrinsically, always show more disability than men at a population level [41]. Exploration of the biological mechanisms underlying the sex differences may assist our understanding and point the way to interventions to prevent or ameliorate the disabling effects of diseases.

By age 85 women have significantly more disability and disabling diseases such as arthritis and hypertension. Whilst hypertension may be asymptomatic, its potential sequelae such as ischemic heart disease, heart failure and CVA could be the driving forces behind its disabling effects. Although men have slightly, though not significantly, more fatal diseases (cardiac disease and CVD), women with these diseases, as well as with respiratory disease, are more likely to become disabled. Men without disability are significantly more likely to die from cancer and respiratory disease but all other transitions from a disabled or non-disabled state were similar for men and women. In addition, we found that overall disability was more of a risk factor for male mortality (disease count adjusted) compared to women and is supported by recent findings [42].

We conclude that the disability-survival paradox is still evident in the very old and appears due to sex differences in the types and impacts of disease.

## Supporting Information

Table S1 Disease duration (years) by sex – median (IQR).
(DOCX)
