# Supplementary material for: The Contribution of Diseases to the Male-Female Disability-Survival Paradox in the Very Old: Results from the Newcastle 85+ Study
Source: PLoS One. 2014 Feb 7;9(2):e88016. doi: 10.1371/journal.pone.0088016 (PMC3917849; doi:10.1371/journal.pone.0088016)
Supplement: Table S1 — Disease duration (years) by sex – median (IQR). (DOCX) [file pone.0088016.s001.docx]

**Table S1: Disease duration (years) by sex – median (IQR)**

|  | Men | Women | All | Sex difference p-value^*^ |
| --- | --- | --- | --- | --- |
| Arthritis | 16 (8-25) | 16 (8-25) | 16 (8-25) | 0.789 |
| Hypertension | 10 (5-18) | 9 (5-15) | 9 (5-16) | 0.274 |
| Cardiac Disease | 12 (8-19) | 10.5 (6-16) | 11 (6-18) | 0.135 |
| Cerebrovascular Disease | 7 (4-12) | 6.5 (3-12) | 7 (3.5-12) | 0.515 |
| Respiratory disease | 13 (5-20) | 12 (6-20) | 12 (6-20) | 0.786 |
| Diabetes | 7 (4-12) | 8.5 (5-15) | 8 (4.5-13.5) | 0.372 |
| Cognitive Impairment^†^ | - | - | - | - |
| Cancer | 1 (1-3) | 1 (1-3) | 1 (1-3) | 0.503 |

^*^Mann-Whitney U Test for gender difference in disease duration

^†^Assessed at baseline therefore no duration
